# Hand Motion Catalog of Human Center-Out Transport Trajectories Measured Redundantly in 3D Task-Space

**DOI:** 10.1038/s41597-025-05576-7

**Published:** 2025-07-24

**Authors:** Tim Sziburis, Susanne Blex, Tobias Glasmachers, Ioannis Iossifidis

**Affiliations:** 1 Institute of Computer Science, Ruhr West University of Applied Sciences, 45479 Mülheim an der Ruhr, Germany; 2 Institute for Neural Computation, Ruhr University Bochum, 44801 Bochum, Germany; 3 Faculty of Electrical Engineering and Information Technology, Ruhr University Bochum, 44801 Bochum, Germany; 4 Faculty of Physics and Astronomy, Ruhr University Bochum, 44801 Bochum, Germany

**Keywords:** Biomedical engineering, Physiology, Motor control

## Abstract

Motion modeling and variability analysis bear the potential to identify movement pathology but require profound data. We introduce a systematic dataset of 3D center-out task-space trajectories of human hand transport movements in a standardized setting. This set-up is characterized by reproducibility, leading to reliable transferability to various locations. The transport tasks consist of grasping a cylindrical object from a unified start position and transporting it to one of nine target locations in unconstrained operational space. The measurement procedure is automatized to record ten trials per target location and participant. The dataset comprises 90 movement trajectories for each hand of 31 participants without known movement disorders (21 to 78 years), resulting in 5580 trials. In addition, handedness is determined using the EHI. Data are recorded redundantly and synchronously by an optical tracking system and a single IMU sensor. Unlike the stationary capturing system, the IMU can be considered a portable, low-cost, and energy-efficient alternative to be implemented on embedded systems, for example in medical evaluation scenarios.

## Background & Summary

The experimental observation and analysis of reaching is a key aspect in developing a systematic understanding of movement generation. As a specific form of a reaching task (also referred to as aiming task), a transport (or transportation) task can be understood as the combination of a first reach-to-grasp movement, the grasping of the object itself, followed by transporting the object and reaching towards a target before positioning it at the target location. In this study, we measured the movement trajectories during the time of transporting the grasped object, i. e. without considering grasping activity.

Over the last decades, there have been various studies in the discipline of neuroscience comprising different variants and phases of reaching task experiments. Studies with human participants include analyses of kinematic coordination and muscle activity, such as regarding trial consistency and coupling between distal and proximal kinematic chain trajectories^1^, or with a specific focus on three-dimensional target positioning^2^. Furthermore, there are studies on hand selection strategy^3^, decision-making^4^, and motor sequence learning^5^. Reaching experiments were also conducted with non-human primates: Georgopolous *et al*. described the activation of specific direction-coded neural populations during two-dimensional^6^ and three-dimensional^7,8^ reaching tasks.

Nevertheless, datasets of the experiments are sparsely publicly available, do not specifically target object transportation, or inquire about 2D movements with touchscreens or 3D movements in virtual reality.

Several motion-captured records of entire body movements can be found in the Carnegie Mellon University Motion Capture Database^9^. This comprises complex data of 144 subjects in human-human and human-environment interaction, locomotion, activities of daily living, as well as physical exercises. However, sufficient systematic data are not included for reaching or transporting in a standardized setting.

A similar approach follows the KIT Whole-Body Human Motion Database^10^, but further introduces a human reference model of kinematics and dynamics. With that, it provides different datasets on normalized motion of human participants and objects they interacted with in constrained and unconstrained environments. While most of the data were recorded via passive optical motion capture, a subset regarding bimanual manipulation^11^ contains multi-modal data (optical, IMUs, depth and standard cameras, data gloves) from two participants.

Another collection of published datasets is gathered in the Database for Reaching Experiments and Models (DREAM) from the Northwestern University^12^. In this database, various datasets captured from reaching tasks are published^13^ together with specific tools and models, expressing a special focus on neural activity. Conducted with different modalities of recording, it comprises datasets of reaching studies on:

- observations of reaching tasks without primary motor cortex population vectors pointing in hand motion direction, measuring neural activity in monkeys^14^,
- probabilistic models during sensorimotor learning via optical finger tracking in the plane^15^,
- neural tuning of movement space in environments of differing complexity, measured by grasping a robotic arm exerting perturbing forces to change movement direction^16^,
- generalization of dynamics learning to novel directions by interpolation, horizontally measured by optical encoders of a robotic handle also exerting forces^17^,
- movement timing and speed-accuracy trade-off (Fitt’s law), measured with a stylus on a tablet^18^,
- nervous system motor adaptation to errors, proposing a non-linear model, recorded by a robotic manipulandum^19^,
- influences of perturbations, measured by moving a horizontally restricted robot arm^20^,
- force production and generalization with differing movement amplitudes while moving a robotic manipulandum^21^,
- multi-sensory integration while moving a haptic device in a horizontal manner and also measuring electrooculography^22^,
- effects of motor-learning on somatosensory plasticity, experiments conducted with a planar robotic arm handle with optical encoders and force-torque sensors for measurements^23^,
- tuning curve stability of neural representation of limb motion during center-out reaching of monkeys, measured from primary motor cortex^24^,
- changes of neural functional connectivity after motor learning, measured by a planar robotic handle together with MRI scans^25^,
- neuroprosthetic control by recording a stylus attached to a robot in combination with eye- and head-tracking as well as electromyography^26^,
- the effect of uncertainty on the generalization of visuomotor rotations by optical tracking a finger used for two-dimensional cursor control^27^, and
- motion target and trajectory decoding of center-out and random target tasks from neural recordings of monkeys, moving and tracking a two-link manipulandum^28^.

However, none of these studies specifically examines the object transport after grasping, which is the target of this study.

Several hand motion datasets were made public via Nature’s Scientific Data. These include movement data with regard to:

- activities of daily living^29–33^
- hand poses^34^ and detailed hand movements^35^ using a wearable glove,
- pick-and-lift and pick-lift-and-move, again using a wearable glove^36^,
- gestures via a redundant set-up of IMUs and cameras^37^,
- and pick-and-place movements in virtual reality^38^.

In comparison to these described experiments, in the present study, the transportation movement using a physical object is captured exclusively. This enables the identification of minor differences in the execution of movement between the participants, and in the future, compared to diagnosed movement disorders. Furthermore, understanding movement generation promises to be more successful for the examination of a single movement phase.

Experiments involving data from actual transport tasks with healthy participants consisted of studies regarding force trajectories^39^, correction motion in the presence of moving targets^40^, or age-dependent motor coordination^41^. Several transport motion analyses with participants suffering from diseases captured data in comparison to healthy control groups. These included the examination of force control in Huntington’s disease^42^, movement dexterity of Parkinson’s disease patients^43^, or eye-hand coordination under hemiparetic cerebral palsy^44^. These studies targeted specific pre-selected movement properties that were supposedly characteristic of the individual use case and lacked the systematic variation of the set-up, such as regarding target positioning. Therefore, the data from these studies, if available, would be limited in terms of detailed and systematic insights into the general processes and methodology behind movement generation.

With specific regard to systematic hand transportation experiments with varying parameters, Grimme *et al*. (2012)^45^ recorded natural human arm motion for two target directions of transporting an object over varying distances as well as obstacle heights and positions. While this first experiment was conducted with ten healthy participants, a second experiment with five participants (again, without known movement disorders) also included further obstacle configurations of one target direction. They analyzed specific invariants and properties of such motion, including isochrony, planarity, and the decomposition of movements into a transport and a lift/descend primitive. Based on extended datasets gathered in further experiments^46^, they additionally describe an obstacle-dependent primitive. A subset of that data was published^47^.

Besides general public availability, our novel dataset provides systematicity with regard to target positioning and consists of methodologically captured hand transport movements in 3D within a simple task set-up. Furthermore, to our knowledge, it is the first dataset comprising transport trajectory recordings from both hands with target-randomized trial settings. The center-out setting of the task can provide information on direction-dependent motion components and factors. The general framework makes it relevant for movement modeling as well as for comparison with later studies on movement impairments.

Usually, optical systems are considered as precise state-of-the-art techniques for motion capture. However, they are typically not suitable for embedded applications: Instead, portable sensors such as inertial measurement units (IMUs) may be favorable, for example, in medical diagnosis scenarios requiring flexible applicability and mobility. Thus, our dataset consists of redundantly measured and synchronized data from both types of systems. In this way, the precision of a portable system can be evaluated with regard to its general appropriateness for varying applications.

Besides the mentioned studies, a limited proportion of previous data publications included more than one system for synchronous measurements. A recent set of data from a redundant approach^48^ combined a set-up of five IMU sensors with active optical motion capture, considering 16 healthy participants performing upper-body movements. In the related studies^49,50^, specific 3D joint angles calculated from the IMUs after different sensor-to-segment calibration methods were validated against the optical reference. The experiment differs from our approach in terms of the motion capture type (active vs. passive), the higher number of IMU sensors, and, accordingly, the IMU calibration procedure, as well as the output variables (joint space vs. task space). Correspondingly, the selected tasks (e. g. elbow flexion, shoulder abduction, drinking) vary from our fundamental transport target variation.

The transport motion data provided in this paper can serve as a basis for the detection of pathological movements by studying movement variability (Sziburis *et al*. in preparation) and identifying individual motion characteristics^51^. In the future, we plan to complement the motion data from healthy participants described in this paper with a dataset of persons experiencing movement disorders, which will be captured in the same way. When combined and compared with such data, our work contributes to modeling the differences between healthy and pathological movement stages. For instance, this can be utilized for the diagnosis of movement disorders or the evaluation of rehabilitation progress.

## Methods

### Ethics Statement

All methods were performed in accordance with the relevant guidelines and regulations, such as the World Medical Association Declaration of Helsinki. The procedures that involve human participants were approved by the Ethics Committee of the Faculty of Medicine at the Ruhr University Bochum (registration number 21-7217). Each participant was informed about the experimental process beforehand and provided written informed consent.

### Participation

The experiments were conducted in November and December 2022 in the eHealth laboratory of the Ruhr West University of Applied Sciences. Experimental data were recorded from thirty-one participants without known movement disorders. After getting introduced to the experimental procedure and providing informed consent, the participants filled out a questionnaire with the following basic information: • age, • gender, • body height, • general physical condition, and • former experience with motion capture experiments.

Furthermore, they conducted the Edinburgh Handedness Inventory^52^ (EHI) in order to calculate their handedness score. The following aspects were addressed: • writing, • painting, • throwing, • scissors, • tooth brush, • knife (without fork), • spoon, • broom (upper hand), • lighting a match, • opening the lid of a box, • preferred foot, and • preferred eye.

The preferred side for each aspect had to be assessed by either two points for a strong preference (the other side not used unless forced) or one point for a weaker preference. If there was no preference for any side, both were rated by one point. The EHI laterality index was then calculated via the point sums for each side (L/R) via:

$$EHI=\frac{R-L}{R+L}\cdot 100$$

**Fig. 1:**
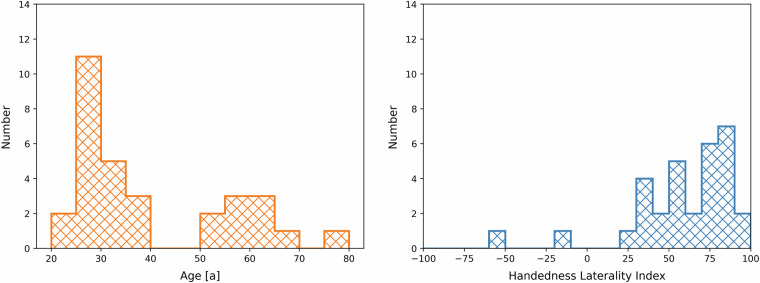
Overview of age and handedness of the study participants. *Left:* Histogram of the study participants’ ages. *Right:* Histogram of the study participants’ handedness laterality indices (EHI scores).

The participants, 11 female and 20 male, were in a range of ages between 21 and 78. Figure 1 shows histograms of age and handedness of the study participants. Age and EHI information are also given in the data themselves, see section Data Records.

### Experimental Design

**Fig. 2:**
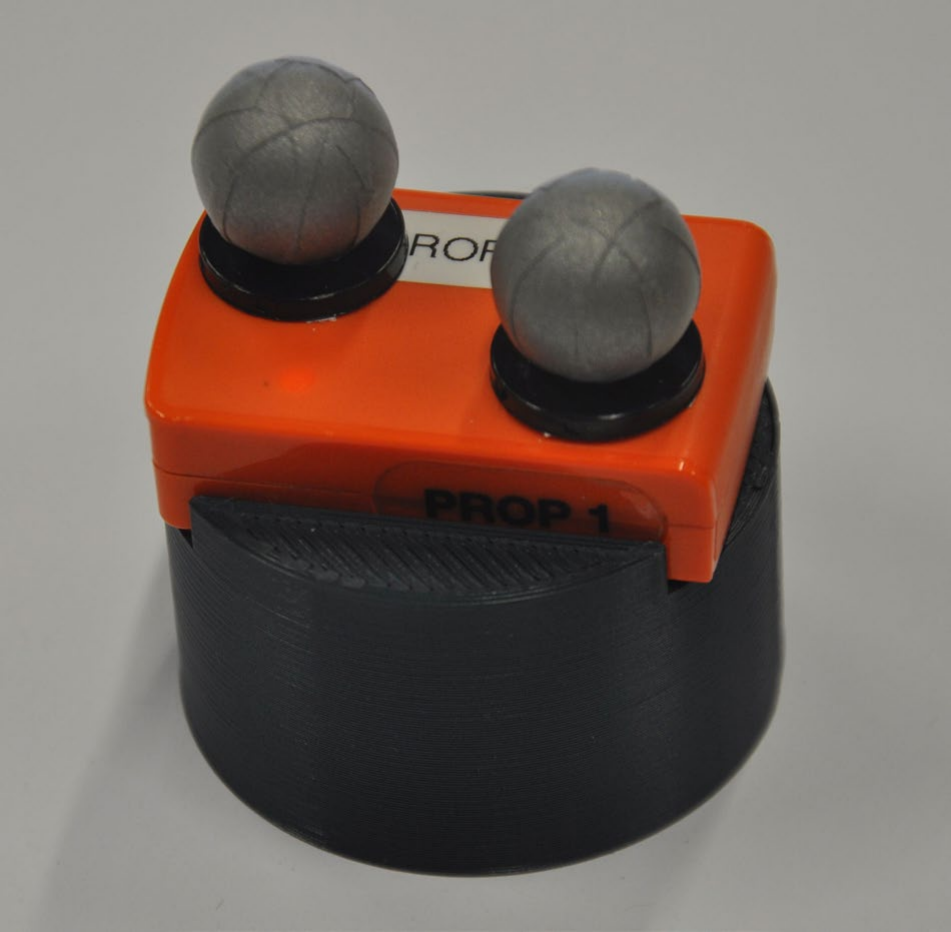
Cylindrical transport object with the IMU sensor on the top, fixed by latches. Two reflective markers for the optical motion capture system are furthermore attached on the top.

Transport trajectories were measured by means of two motion capture systems in parallel, while participants moved a cylindrical solid body from a start position to one of nine target positions. The cylindrical object had a diameter of 5 cm and a height of 2.5 cm. Being individually 3D-printed, the cylinder provided the possibility to lock the used sensor object by latches, see Fig. 2.

In this way, a single inertial measurement unit (IMU) sensor was attached to the cylindrical transport object in order to capture movement data. Additionally, six cameras were positioned in the room to record two reflection elements. These reflective spheres for optical capturing were glued at the top of the IMU sensor diagonally so that at least one of them was always visible from any perspective angle.

Both systems were time-synchronized via the Network Time Protocol (NTP). The cameras were positioned in the room in a way that occlusions caused by the participants’ movements were avoided. Furthermore, the positions of the cameras, their aperture, focus, and zoom settings were adjusted so that the tracking object was recognizable from all cameras and that no reflection disturbances from other sources appeared. For this reason, the windows of the laboratory were shaded, and ceiling lighting was switched on to guarantee identical illumination conditions for all participants.

The IMU data communication base station was positioned at one corner of the table and aligned with its edges, not disturbing or distracting the participants. The experimental location in the laboratory room was selected in a way that magnetic field perturbations influencing the IMU sensor were minimized.

**Fig. 3:**
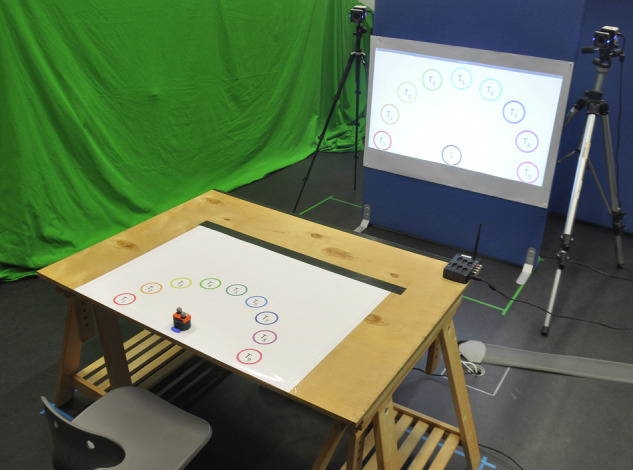
Experimental set-up. While seated, the participants were asked to perform 3D transport tasks from the start to randomized target locations after an acoustic start signal, visually announced in the projection.

**Fig. 4:**
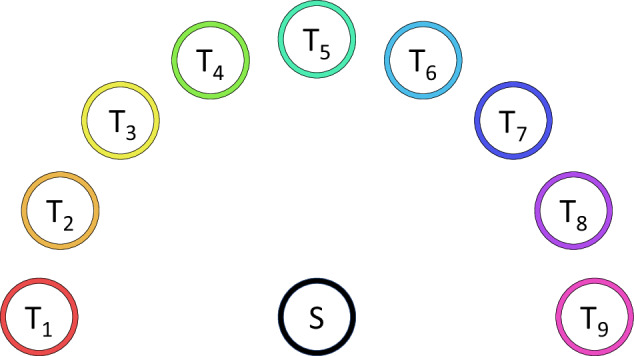
Positioning of the transport targets.

Participants were seated on a chair in front of a table, both of which were adapted to their individual body dimensions (see also section Data Acquisition Workflow). The table was made of wood to further reduce electromagnetic influences. Chair and table positions were fixed in the room; adjustments were possible via height and tabletop position, respectively. On the table, the start position and the target positions were marked by circles, see Fig. 3. The targets were equidistantly placed on a semicircle with a radius of 25 cm as shown in Fig. 4. The start location was in the center of the semicircle. The start and target circles themselves had a radius of 6 cm. The center of the start circle was positioned at 6 cm from the edge of the table. A 3D-printed docking block in the form of a short segment of a circle was glued to the printed starting circle (blue block in Fig. 3). With that, an identical start position could be guaranteed for all trials.

For displaying target cues, a projector was positioned at an unobservable position behind the table, so that the participants experienced no distraction. The projection was displayed on a canvas further in front and was directly visible. Auditory start and stop signals were played via a loudspeaker integrated in the projector.

To avoid rhythmic movement patterns and specific time dependencies such as anticipatory behavior^53^, random delays were introduced between the visual target cue and the acoustic start signal.

Each participant performed two sessions, one for each hand. Half of the participants started with the left-hand session, the other with the right-hand session. Both sessions were performed one after another, separated by a small break of some minutes.

Combining the transport cylinder with the IMU sensor and the two reflective elements for optical tracking, the whole transport object had a weight of 41 g in total.

### Instrumentation

**Fig. 5:**
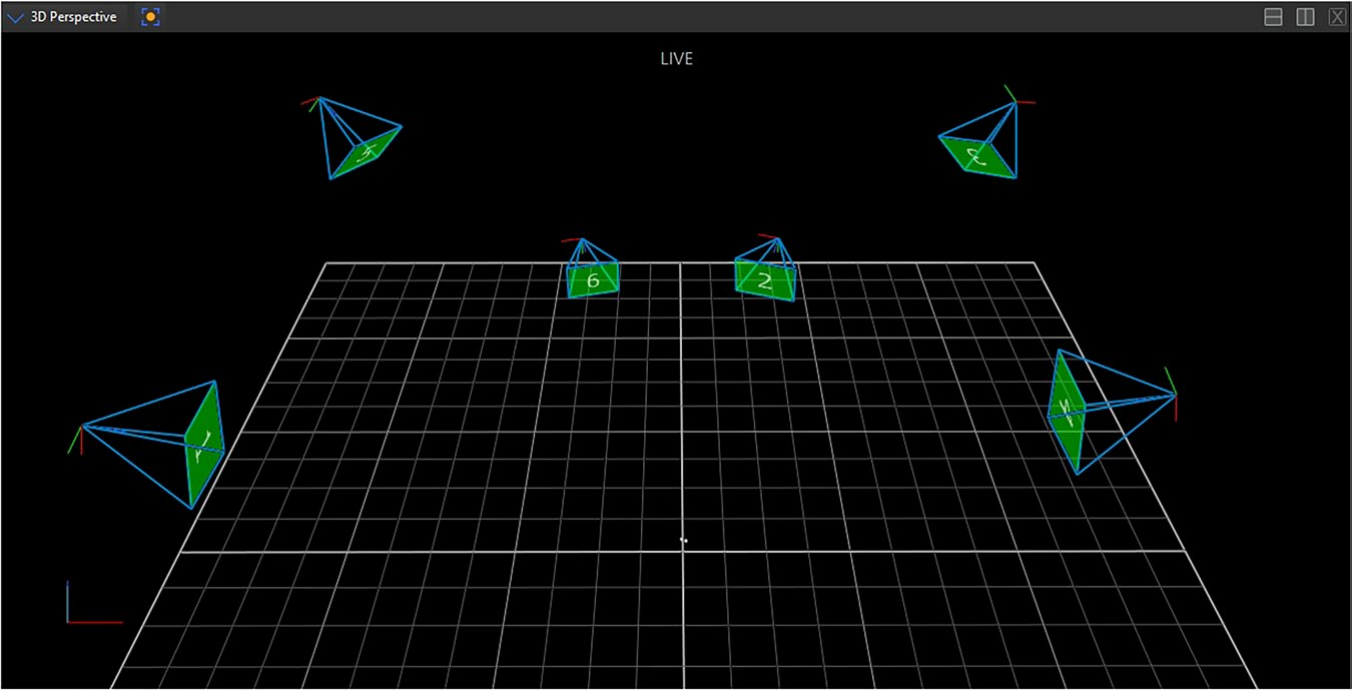
Positioning of the Vicon Nexus optical motion capture cameras in the laboratory room, illustrated in the reconstructed world model. Moreover, the two reflection markers are visible (two small dots near the coordinate origin), which are attached to the cylindrical transport object, located at the start location.

A state-of-the-art optical motion capture system (Vicon Nexus 2.14) comprised of six infrared cameras (Vicon Vero 1.3, with 1.3 MP resolution, maximum frame rate 250 Hz) was utilized to provide precise position reference data. These movements were measured by the continuous optical tracking of two reflection elements attached to the cylindrical transport object. From the infrared recordings of the cameras that were able to detect the reflection elements in each frame, the individual three-dimensional positions were calculated in a world frame and combined into a course of positional data. The capturing rate was configured to the maximum possible frame rate of 250 Hz. The camera positioning is visualized in Fig. 5.

The cameras and the measurement computer of the optical motion capture system were interconnected via RJ45 Ethernet ports. The measurement PC for the optical system was running a 64-bit Microsoft Windows 10 operating system with a 10 Gbit Ethernet controller.

The second measurement system, based on drift-corrected accelerometer data of a single state-of-the-art inertial measurement unit (IMU) from an Xsens MTw Awinda system, was used in parallel to evaluate the quality of a portable sensor set-up with embedded applicability. The following movement data were recorded by the IMU: accelerations (accelerometer), angular velocities (gyroscope), and orientations (magnetometer).

For capturing the IMU data, the Xsens SDK from the Xsens MT Software Suite 2021.4 was utilized. Since an Xsens MTw system was used as opposed to the more recent MTi systems, the closed-source API with proprietary libraries had to be called instead of the open-source API. A C++ program was written that incorporated communication with the Xsens Awinda v2 base station, addressing the single sensor (MTw2), as well as initiating the measurement and eventually recording the data. Both the base station and the sensor were running the most recent firmware (4.6.0).

The base station was connected via USB to a measurement laptop running a 64-bit Debian GNU/Linux 11 “Bullseye”. In order to take advantage of real-time capabilities, a specific kernel was loaded, namely Liquorix 6.0 with preemptive scheduling.

Based on an example for the utilization of the Xsens SDK, the recording program was extended by a Qt graphical user interface to allow the configuration of the communication channel, frame rate, and connected sensor. Moreover, this was applied to configure the experimental control and finally conduct the experiments, including visualizations and acoustic signals. Besides the actual data recordings and their exact start and stop timestamps for all trials, this program calculated and stored the randomized order of targets to reach as well as the durations of all randomization time periods.

### Data Acquisition Workflow

The participants were introduced to the study and received information on the experimental procedure as well as the purpose of the measurements. After they had the possibility to request clarification and details, they were asked to sign an informed consent form. In case of consent, they also filled out the questionnaire described in section Participation.

First, the wooden table and chair were adjusted for each individual participant in a way that they were sitting in an upright position. Furthermore, it was ensured that there was an angle of 90^∘^ inside the elbow while positioning their hand palms on target 1 (the outer left) and target 9 (the outer right). Each participant’s configuration (chair height and tabletop position) was noted.

After that, a calibration of the Vicon Nexus optical motion capture system was initiated via the active wand calibration method, according to the official instructions from the manufacturer, that is, moving the wand evenly in and over all directions of the experimental space until each camera reached a count of 2000 calibration frames. Until a self-set world error threshold could be reached, the procedure was repeated. This was followed by masking the infrared cameras via the Vicon Nexus software in order to eliminate any potentially remaining externally disturbing reflections out of the particular region of measurements.

After successful calibration, the origin of the coordinate frame was set for the Vicon system by means of positioning the calibration wand horizontally on the table with the origin in the center of the starting circle.

We made sure that the battery of the IMU sensor was charged to at least 90% and then continued with magnetic field mapping of the IMU via the corresponding software tool from the MT Software Suite 2021.4 (Magnetic Field Mapper). For this, the IMU sensor, attached to the cylindrical transport object, had to be uniformly rotated around any possible axes, until the criteria for 3D calibration stated in the Xsens manual were fulfilled (for details, see section Technical Validation).

Before seating, the participants were asked to remove all electronic devices and metallic objects from their pockets and from their clothing to minimize electromagnetic disturbances. The task space was systematically adjusted to their individual body dimensions. As soon as they were correctly seated, they had the opportunity to start a prototypical test trial to familiarize themselves with the visual projection, the timing characteristics with randomization, and the start and stop sounds.

**Fig. 6:**
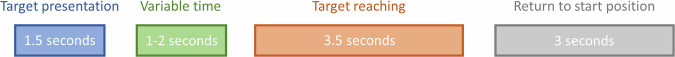
Experimental workflow and measurement sequence of each trial with time periods.

The recording procedure was automatized. The experimental sequence with the corresponding time spans per trial is depicted in Fig. 6.

Each participant performed two experimental sessions, one for each hand. In each transportation task, they grasped the object and moved it from the start to a target position on the labeled table. They were instructed to perform this movement as fast and as precisely as possible, as it is common in literature and described for movements with spatial constraints^54^.

While the start position was always the same, the target position randomly varied between nine possibilities and was visually announced before an acoustic start signal appeared. The time between the visual cue and the acoustic start signal was randomized between one and two seconds. For each target, ten trials were performed for each hand. Conducted as a double-blind study, the appearing targets were neither known to the participants nor to the experimenters beforehand. After finishing a trial, participants had to stay still until an acoustic stop signal appeared, signaling them to move the object back to the starting position again. Between the two sessions, a longer break was scheduled according to their individual needs. Furthermore, the participants could always introduce additional breaks between the trials if needed. No participant requested to make use of this possibility, though. One recording session usually took about 30 minutes (15 minutes per hand).

### Data Processing

While the IMU data capturing program recorded the data on a per-trial basis and not during intermediate time periods, the Vicon system recorded the optical reflection data continuously from the start of the experimental session to its end. After finishing, this data stream was automatically cut into per-trial data via the stored IMU recording timestamps.

For both systems, a rotation was introduced to match the trajectory profiles. The trajectories were rotated in a way that the straight connection line between start and target maps onto the y-axis, calculated individually for each trial.

Since the time synchronization via NTP could have been subject to network delays, an additional fine adjustment of time synchronization was introduced. This precise overall alignment for each trial could be realized via shifting the time courses to match the middle points of position on the y-axis. As both measurement systems provided data at differing sampling rates (250 Hz and 100 Hz), for that time alignment, the trajectories of the IMU system were temporarily resampled to 250 Hz (interpolation through cubic splines). After calculating the offset, it was applied to the original data.

Velocity and acceleration data of the optical system were calculated by differentiation, while for the IMU-based system, velocity and position data were integrated from the acceleration after appropriate preprocessing. A fourth-order Butterworth low-pass filter was applied to all data to remove high-frequency noise from the trajectories (cut-off frequency 7 Hz as a result of prototypical tests).

#### Optical Data

The original data from the optical motion capture system were captured in the proprietary Vicon Nexus file format. After first preprocessing as described in the following, the position data of both markers were exported in the form of CSV files. The optical system utilized the infrared reflection recordings from all cameras, which were able to perceive at least one marker in order to reconstruct the position course of the transported object in a world coordinate frame. For this, both markers had to be labeled in the Vicon Nexus software. If this assignment was not possible in a continuous manner, for example, due to occlusions or the instantaneous appearance of further reflection signals, the labeling had to be performed for each self-contained sequence. If one optical marker was temporarily occluded by a participant’s limb or head, for example, the missing position of this marker in single frames could be padded by pattern fill: Since the two markers had a fixed position relative to each other, the pattern of movement could be inferred from the non-occluded marker. This was automatically manageable in the Vicon software.

If in single frames both markers appeared to be not visible, automatic spline fills (polynomial order 5) were applied, interpolating the previous and the subsequent visible marker positions. This was only applied if there were not more than five missing frames. In the rare case of a larger gap, the particular trial was disregarded.

If there was not only a temporary, but a full occlusion of one marker, only the visible marker’s position recording was taken into account (again, with potential spline fillings for up to five frames).

For calculating the courses of velocity and acceleration from the positional data, the numerical derivatives were derived via finite differences. A central differencing method using three points was applied.

#### IMU Data

The IMU data from the Xsens sensor were saved in the proprietary Xsens format and converted to CSV files. One file was recorded per trial. Furthermore, for each session (left or right hand for each participant), the randomized delays between visual cue and acoustic start signal, the order of targets appearing, and the individual start and stop timestamps of each trial were stored.

Generally, IMU data are often used for orientation estimation. However, for the presented dataset, only the accelerometer data were used for position determination. Measures to compensate for sensor drift needed to be introduced, though. In preliminary tests, the gyroscope and magnetometer data were incorporated as well to calculate estimated corrections of the accelerations by these additionally measured values and the application of an extended Kalman filter. While introducing high computational demands, this did not show any improvement in the acceleration data or, after integration, velocity and position estimation, respectively. For this reason, and to apply the least possible preprocessing but as much as necessary, the extended Kalman filter approach was eventually not applied. The finally conducted steps are described in the following.

At first, for the acceleration data, the free acceleration needed to be calculated. For this, the gravitational acceleration could be subtracted from the measured acceleration data. The gravitational acceleration could be determined during the resting state without any external movement. The free acceleration was also provided by the embedded processing of the utilized IMU sensor itself.

For acceleration drift compensation, movement initiation was defined as the time point when an empirically determined acceleration noise threshold, combining all three dimensions of the acceleration vector in the form of Euclidean norm, is exceeded. For this, the remaining noise acceleration during the resting state after the end of the movement was utilized, precisely the per-component maximum of the last ten samples.

The acceleration values during initiation and ending resting state (phases under the acceleration threshold) were corrected by subtracting the averaged noise activity. The averaged acceleration sensor drift rate was then calculated between movement initiation and end. With this drift rate, the intermediate acceleration measurements at each time point during movement could be corrected by the relative proportion of overall drift.

To implement the integration of acceleration to obtain velocity, an approach related to drift correction was followed, called zero velocity update (ZUPT), originally proposed for navigation and survey applications^55^.

Following this method, the acceleration time course was split into continuous segments of under-or-equal-threshold and over-threshold activity, again depending on the same empirically determined acceleration threshold. For all samples, a correction of velocity due to drifting sensor data was applied, while during the phases of under-threshold activity, the velocity was reset to zero. This could prevent the integrative accumulation of drift errors.

First, the velocity was calculated kinematically as if there was no drift: *v*_*t*+1,*pred*_ = *v*_*t*_ + *a*_*t*_ ⋅ *dt*.

For the first sample of an under-threshold phase, the velocity drift rate between the current sample and the last sample of the previous under-threshold phase was calculated by dividing their velocity difference by time (sample count). Ideally, in these samples, the acceleration activity would be zero if there were no drift or noise. Within the under-threshold phases (particularly including the very beginning of the movement, where no previous under-threshold phase exists), the velocity was set to zero. With the drift rate, the predicted velocity values of the over-threshold activity phase lying directly in between the under-threshold phases were corrected by subtracting the corresponding drift in order to guarantee continuity.

In principle, zero acceleration could also mean arbitrary constant velocity instead of zero velocity. However, this was practically not relevant due to highly variable acceleration courses and the accelerometer’s sensitivity at close-to-constant velocities. This led to accelerometer responses even to low deviations, such as noise.

Finally, with the zero-velocity-updated integration of acceleration to obtain the course of velocity, the position course was kinematically computed based on these corrected velocity values: ${s}_{t+1}={s}_{t}+{v}_{t}\cdot dt+\frac{1}{2}\cdot {a}_{t}\cdot d{t}^{2}$.

## Data Records

The dataset is published via the Open Science Framework (OSF.io) platform^56^.

After extracting the data archive, within the Output directory, the captured records can be found in the form of CSV files, structured in the three main directories measurement, processed, and rotated. Within these three directories, there is a folder structure naming the number of the participant, the hand recorded (left/right), and the capturing system used (IMU: Xsens, and optical: Vicon, respectively), e. g. for subject 23: 23/L/X, 23/L/V, 23/R/X, and 23/R/V.

The general contents of the main folders are presented in the following (the session ID [SesID] is composed of the subject number in the interval [1, 31], sorted by age, and the recorded hand in {L, R}):

1. measurement for the recorded data without further processing (for Vicon, the data are just split into single files and preprocessed by Nexus software):
  ordered list of all targets in the session:
    [SesID]_targets.txt,
  ordered lists of recording timestamps for all trials’ start and end times in the session:
    [SesID]_timestampsRecStart.txt and
    [SesID]_timestampsRecStop.txt, respectively
    (format YYYY-MM-DD-hh-mm-ss.sss), as well as
  ordered list of the added randomized time delays for all recording trials in the session:
    [SesID]_timeDelays.txt, in milliseconds,
  positions for Vicon data,
    CSV file [SesID]_V_rec[0,89].csv:
    frame number (1 column),
    {x,y,z} positions of marker 1 [mm] (3 columns),
    {x,y,z} positions of marker 2 [mm] (3 columns).
  IMU values for Xsens data,
    CSV file [SesID]_X_rec[0,89].csv:
    {x,y,z} acceleration [ms^−2^] (3 columns),
    {x,y,z} free acceleration [ms^−2^] (3 columns),
    gyroscope values (angular velocities [rad s^−1^], 3 columns),
    magnetometer raw values (arbitrary unit, normalized to calibration magnetic field strength, 3 columns),
    rotation quaternion (unit-free, 4 columns: 1 real part, 3 imaginary parts).
2. processed for the measurement data processed by:
  filtering as described above,
  correction for acceleration data (drift, ZUPT),
  time alignment as mentioned above, as well as
  differentiation of positional Vicon data to derive velocity and acceleration,
    CSV file [SesID]_V_rec[0,89].csv:
    time [s] (1 column),
    {x,y,z} positions [m] (3 columns),
    {x,y,z} velocities [ms^−1^] (3 columns),
    {x,y,z} accelerations [ms^−2^] (3 columns),
  and integration for IMU accelerometer data to calculate velocity and position,
    CSV file [SesID]_X_rec[0,89].csv:
    time [s] (1 column),
    {x,y,z} positions [m] (3 columns),
    {x,y,z} velocities [ms^−1^] (3 columns),
    {x,y,z} accelerations [ms^−2^] (3 columns).
3. rotated for the final rotated data, i. e. after applying to the processed data:
  rotation to map the straight positional start-target-line onto the y-axis for each trial,
  leading to the same CSV columns as in the case of the processed data,
    CSV files: [SesID]_V_rec[0,89].csv for Vicon, and
    [SesID]_X_rec[0,89].csv for Xsens.

Furthermore, on the top directory level, there is a Readme.txt file with information on how to use the dataset. On the same level, the text file ExcludedTrials.txt provides information on which trials to exclude in analyses due to data quality issues. The anonymized CSV file ParticipantInformation.csv includes the ages of participants and their EHI scores, based on the questions asked beforehand. If desired, the Python preprocessing scripts preprocess_IMU.py and preprocess_optical.py, respectively, can be found within the Code directory.

### Example Data Record Structure

As an example, Table 1 illustrates the directory and file structure of the extracted dataset archive for subject number 1 (within the Output folder). Subject numbers in the interval [1, 31] can be chosen. The number of lines of the movement data files depends on the individual recordings and the particular measurement system (250 Hz capturing rate for Vicon, 100 Hz for Xsens).

**Table 1:** Structured data for subject number 1 as an example (within folder Output).

Path	File	Lines	Description
measurement/1/L	1_L_targets.txt	90	Ordered list of target positions
measurement/1/L	1_L_timeDelays.txt	90	Ordered list of added delays [ms]
measurement/1/L	1_L_timestampsRecStart.txt	90	Ordered list of recording start times
measurement/1/L	1_L_timestampsRecStop.txt	90	Ordered list of recording end times
measurement/1/L/V	1_L_V_rec[0,89].csv	#frames (@250Hz)	Positions of both markers
measurement/1/L/X	1_L_X_rec[0,89].csv	~3.5*s@*100*Hz*	IMU values (see above)
measurement/1/R	1_R_targets.txt	90	Ordered list of target positions
measurement/1/R	1_R_timeDelays.txt	90	Ordered list of added delays [ms]
measurement/1/R	1_R_timestampsRecStart.txt	90	Ordered list of recording start times
measurement/1/R	1_R_timestampsRecStop.txt	90	Ordered list of recording end times
measurement/1/R/V	1_R_V_rec[0,89].csv	#frames (@250Hz)	Positions of both markers
measurement/1/R/X	1_R_X_rec[0,89].csv	~3.5*s@*100*Hz*	IMU values (see above)
processed/1/L/V	1_L_V_rec[0,89].csv	#timesteps (@250Hz)	Pos., vel., acc. (filtered, aligned)
processed/1/L/X	1_R_X_rec[0,89].csv	#timesteps (@100Hz)	Pos., vel., acc. (filtered, aligned)
processed/1/R/V	1_L_V_rec[0,89].csv	#timesteps (@250Hz)	Pos., vel., acc. (filtered, aligned)
processed/1/R/X	1_R_X_rec[0,89].csv	#timesteps (@100Hz)	Pos., vel., acc. (filtered, aligned)
rotated/1/L/V	1_L_V_rec[0,89].csv	#timesteps (@250Hz)	Pos., vel., acc. (additionally rotated)
rotated/1/L/X	1_L_X_rec[0,89].csv	#timesteps (@100Hz)	Pos., vel., acc. (additionally rotated)
rotated/1/R/V	1_R_V_rec[0,89].csv	#timesteps (@250Hz)	Pos., vel., acc. (additionally rotated)
rotated/1/R/X	1_R_V_rec[0,89].csv	#timesteps (@100Hz)	Pos., vel., acc. (additionally rotated)

### Example Trajectory of Processed and Rotated Recording

**Fig. 7:**
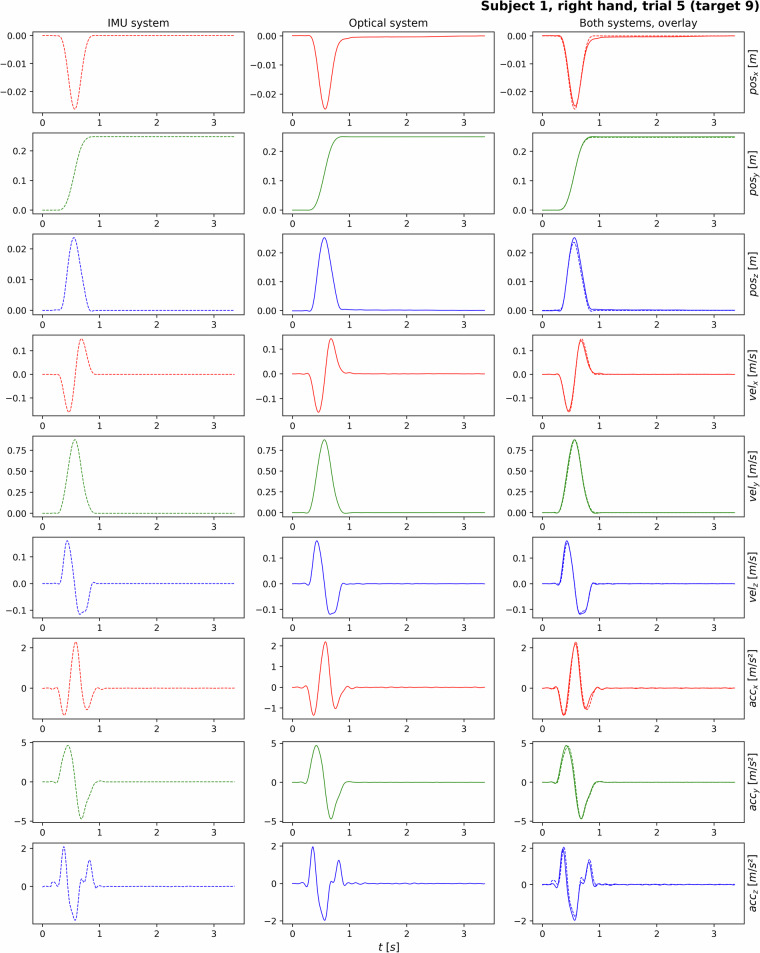
Processed and rotated trajectory, exemplary recording of one subject, visualizing position, velocity, and acceleration for the individual dimensions over time [s]. The first two columns show the data of the individual measurement systems, the third an overlay of both systems’ data.

An exemplary trajectory of the processed and rotated data can be seen in Fig. 7, i. e. after filtering, drift correction, and fine alignment. This depicts one trial’s position, velocity, and acceleration courses over time (in seconds) for the x, y, and z dimensions of subject 1 (trial 5). While the first column shows the data from the optical system and the second column that from the IMU system, the third visualizes the overlay of both systems.

## Technical Validation

The Vicon Nexus motion capture set-up, mentioned as the optical reference measurement system, was calibrated with the Wand calibration method recommended by the manufacturer. At least 2000 calibration frames were gathered per camera, until a maximum world error of 0.17 mm per camera could be guaranteed by the software. To further reduce errors and provide redundancy, two reflection elements were attached to the cylindrical object, maintaining a fixed distance from each other. This made sure that occlusions of both markers at the same time could be avoided. When both markers were detected, the position of the transport object was determined by the mean of the two markers’ positions, further minimizing noise effects. In the case that, for a short time, only one marker was visible to sufficiently many cameras to determine its position, the positional course of the other could be reconstructed by the software’s functionality of pattern filling for single frames, orienting to the movement behavior of the visible marker.

As IMU sensors are known to be prone to disturbances, at first, a pilot study was conducted in an electrically shielded lab to avoid the effect of electrostatic discharge. This analysis could not reveal any apparent difference in data quality when compared to the standard, non-shielded laboratory environment where the optical system was available. Inside the non-shielded laboratory, magnetic field mappings were conducted by means of the Xsens Magnetic Field Mapper software to find the most suitable location for the experimental setup and to calibrate the sensor concerning the locally prevailing magnetic field. The software provided different criteria for the quality of calibration by deriving a model mapping the measured magnetic field to an ideal sphere with center at zero and a vector magnitude of 1: average of the magnetic norm close to 1, maximum error with respect to a norm of 1 and standard deviation of the norm as low as possible, no large spikes in spatial distribution of difference, and residuals of the corrected magnetic field vector following a Gaussian distribution^57^.

The laboratory location with the least temporal and spatial disturbances during the calibration process with the Magnetic Field Mapper was chosen for the experiments. Moreover, before each experimental session, further magnetic field mappings were undertaken to consider the effect of static disturbances, i. e. to predict and compensate for deterministic, constant magnetic field errors. As suggested by the manufacturer’s manual for 3D calibration, the IMU sensor was rotated in as many orientation angles as possible at the same location (no translational velocity) with an approximately constant angular velocity during about three minutes of magnetic field mapping.

Additionally, before each session, it was made sure that the battery of the IMU sensor was always fully charged with at least 90%, so that voltage-descent-dependent effects could be excluded as sources of errors. The wireless IMU communication with the real-time Linux kernel to avoid data loss of single frames due to process scheduling was examined in prototypical tests. These showed that all data could be successfully transferred at a rate of 100 Hz. The utilized transfer protocol is based on the IEEE 802.15.4 standard (low-rate wireless personal area network, LR-WPAN). A fully reliable communication could be established via the Xsens wireless channel 25 (center frequency 2.475 GHz). In comparison to other possible channels, pilot experiments showed that this wireless channel could dependably minimize interference with other devices operating in the 2.4 GHz band (e. g. Bluetooth, IEEE 802.11/WLAN).

After data capturing, all recorded trajectories were checked for errors. Those with a high deviation between the final movement position and the task target (>4 cm) or not heading towards the target at all have to be excluded. Also, the trajectories with an atypical elevation amplitude (>20 cm) or no elevation are to be disregarded. In some cases, participants began moving before the acoustic start signal or tried to correct their movements. Furthermore, data with movement artifacts stemming from IMU acceleration integration or due to non-correctable occlusions of the optical system (missing frames) should not be considered. A sufficient, but not necessary criterion that turned out for these cases of artifacts was a high average of the absolute curve torsion values (>0.5 cm^−1^) during the motion travel (middle) phase, infringing the planarity of movement paths. In the end, a manual error check of all trial trajectories was performed. A list of all trajectories to disregard can be found in the main directory of the data archive (ExcludedTrials.txt).

## Usage Notes

The dataset comprises 31 subjects. The recruitment of participants by personal request does not warrant a representative selection compared to the general population.

For the motion trajectories, some trials to single targets have to be disregarded due to bad quality, as described in the section on Technical Validation. For the optical motion capture, this mainly stems from occlusions. In some cases, those could not be fully avoided, particularly when concerning both infrared reflection markers at the same time. In the case of the IMU system, the accelerometer sensor is sensitive to the exerted velocity. While fast movements, including abrupt changes of accelerations when moving the cylindrical object back onto the table, did not affect data quality, the effect of sensor drift for very slow movements could not be sufficiently compensated. However, since the paradigm was that movements had to be executed as fast and precisely as possible, too slow movements appeared only in rare cases. These individual trials have to be disregarded, too.

## Data Availability

The custom data processing source code is publicly available within the Code directory of the data repository^56^. Custom Python 3 code was utilized for preprocessing: For the IMU system’s data, this comprises filtering, drift correction, and integration of acceleration data. For the optically measured data, the processing code includes filtering, numerical differentiation of the position data, and time synchronization by offset adjustment. In both cases, further custom code was in use regarding trajectory rotation for data comparability. For the step of recording the optical motion capture data, no custom code was used. The standard processing pipelines from Vicon Nexus 2.14 for reconstructing, marker labeling, pattern filling of single missing frames, and data export were applied. The developed C++ code for conducting the overall experiment and recording IMU data was based on an example from the Xsens MT Software Suite 2021.4 software development kit with a graphical Qt user interface.
